# What have we learned about communication inequalities during the H1N1 pandemic: a systematic review of the literature

**DOI:** 10.1186/1471-2458-14-484

**Published:** 2014-05-21

**Authors:** Leesa Lin, Elena Savoia, Foluso Agboola, Kasisomayajula Viswanath

**Affiliations:** 1 Department of Social and Behavioral Sciences, Harvard School of Public Health, 677 Huntington Avenue, Landmark Center, 3rd Floor East, Boston MA 02115, USA; 2 Dana-Farber Cancer Institute, 450 Brookline Avenue, Boston MA, USA; 3 Department of Biostatistics, Harvard School of Public Health, 677 Huntington Avenue, Boston, MA, USA; 4 Division of Policy Translation and Leadership Development, Harvard School of Public Health, 677 Huntington Avenue, Boston, MA, USA

**Keywords:** Communication, Communication inequalities, Risk communication H1N1, Pandemic, Public health emergency preparedness

## Abstract

**Background:**

During public health emergencies, public officials are busy in developing communication strategies to protect the population from existing or potential threats. However, a population’s social and individual determinants (i.e. education, income, race/ethnicity) may lead to inequalities in individual or group-specific exposure to public health communication messages, and in the capacity to access, process, and act upon the information received by specific sub-groups- a concept defined as communication inequalities.

The aims of this literature review are to: 1) characterize the scientific literature that examined issues related to communication to the public during the H1N1 pandemic, and 2) summarize the knowledge gained in our understanding of social determinants and their association with communication inequalities in the preparedness and response to an influenza pandemic.

**Methods:**

Articles were searched in eight major communication, social sciences, and health and medical databases of scientific literature and reviewed by two independent reviewers by following the PRISMA guidelines. The selected articles were classified and analyzed in accordance with the Structural Influence Model of Public Health Emergency Preparedness Communications.

**Results:**

A total of 118 empirical studies were included for final review. Among them, 78% were population-based studies and 22% were articles that employed information environment analyses techniques. Consistent results were reported on the association between social determinants of communication inequalities and emergency preparedness outcomes. Trust in public officials and source of information, worry and levels of knowledge about the disease, and routine media exposure as well as information-seeking behaviors, were related to greater likelihood of adoption of recommended infection prevention practices. When addressed in communication interventions, these factors can increase the effectiveness of the response to pandemics.

**Conclusions:**

Consistently across studies, a number of potential predictors of behavioral compliance to preventive recommendations during a pandemic were identified. Our findings show the need to include such evidence found in the development of future communication campaigns to ensure the highest rates of compliance with recommended protection measures and reduce communication inequalities during future emergencies.

## Background

During public health emergencies, public officials are busy in developing communication strategies to protect the population from existing or potential threats. In many situations, such as the early stages of a pandemic, their capability to effectively communicate risk and recommend preventive behaviors may be the only public health strategy to reduce morbidity and mortality [1]. However, a population’s social and individual determinants (i.e. education, income, race/ethnicity) may lead to inequalities in individual or group-specific exposure to public health communication messages, and in the capacity to access, process, and act upon the information received by specific population sub-groups- a concept defined as communication inequalities [2-6]. Such inequalities may lead to disparities across segments of the population in the ability to comply with recommended preventive behaviors [3,7-11]. In 2009, a new strain of H1N1 influenza virus spread around the world causing a new pandemic. The initial outbreak began in the state of Veracruz, Mexico, the Mexican government closed most of Mexico City's public and private facilities in an attempt to contain the spread of the virus [12,13]. However, it continued to spread globally, and in June of the same year the World Health Organization (WHO) declared the outbreak a pandemic [14]. Historically, there has been a paucity of empirical literature in the investigation of social determinants of communication inequalities in emergency preparedness [7]. The H1N1 pandemic offered a unique opportunity to researchers to assess the impact of communication efforts in response to an influenza outbreak of uncertain evolution leading to a robust research program in the field of communications in public health emergency preparedness and risk communication [7].

What scientists have learned about communication inequalities from this experience could help public health officials in identifying what communication strategies work better to prepare the public to respond to future emergencies. The aims of this literature review are to: 1) characterize the scientific literature that examined issues related to communication to the public during the H1N1 pandemic, and 2) summarize the knowledge gained in our understanding of social determinants and their association with communication inequalities in the preparedness and response to an influenza pandemic.

## Methods

### Literature search sources and strategies

Eight databases were systematically searched: PubMed, EMBASE, PsycINFO, Education Resources Information Center (ERIC), Communication Abstracts, Sociological Abstracts, Applied Social Sciences Index and Abstracts (ASSIA), and Web of Science. Literature relevant to the topic of interest, "Public Health Emergency Preparedness (PHEP) Communications during the 2009 H1N1” reported from January 1, 2009 to August 1, 2012 were identified. A list of search strategies and key words used for the databases along with numbers of articles found from each database are reported in Table 1. Articles were considered for inclusion if they addressed issues regarding communications with the public during the H1N1 pandemic and were derived from empirical data. Articles were included if written in in any of the following 6 languages: English, French, Italian, Spanish, Chinese and Portuguese. Two reviewers independently screened articles identified from the initial search by title and/or abstract.

**Table 1:** Search strategies and key words used

**Databases**	**Search strategy**	**Results**	**Title screening**	**Duplicate**	**Full review**
**PubMed**	("Public Health"[All] OR "World Health"[All] OR “Health” [tiab]) AND ("Influenza A Virus, H1N1 Subtype"[Mesh] OR h1n1[tiab] OR swine flu[tiab] OR swine influenza[tiab]) AND ("communication"[Mesh] OR "Health Knowledge, Attitudes, Practice"[Mesh] OR "Access to Information"[Mesh] OR "Communications Media"[Mesh] OR "Internet"[Mesh] OR "J Health Commun" [Journal] OR "Health Commun"[Journal] OR communication*[tiab] OR media[tiab] OR knowledge[tiab] OR information[tiab] OR awareness[tiab]) NOT “therapeutic use*”[All] NOT “reproductive”[All] NOT “records as topic”[Mesh] NOT “exercise*”[ti] NOT “modeling”[All] NOT “modelling”[All] NOT “tobacco”[All] NOT “ethic*”[tiab] NOT “veterinary medicine”[Mesh] NOT “insurance”[ti] NOT “disorders”[tiab] NOT "contracept*"[tiab] NOT "emergency medicine"[Mesh] NOT clinical*[ti] NOT “dental”[All] NOT “stroke”[All] NOT “law”[title] NOT “legal”[ti] NOT “pharmacist*”[tiab] NOT “pharmaceutical*”[tiab] NOT “HIV”[ti] NOT “surveillance” [ti] NOT "emergency department" [tiab] NOT “AIDS”[ti] NOT “provider*”[ti] NOT “worker*”[ti] NOT "departments"[ti] NOT "personnel"[tiab] NOT "nurses"[tiab] NOT "nursing"[tiab] NOT “nurse*”[tiab] NOT "hospital*" [ti] NOT "sales" [ti]	355	148	3	204
	*Filters: Journal Article; Full text available; Publication date from 2009/01/01 to 2012/08/01; Humans; Chinese; Spanish; English; Portuguese; French; Italian*				
**EMBASE**	public health'/exp OR 'public health' OR 'public health':ab,ti OR 'world health':ab,ti AND ('h1n1':ab,ti OR 'swine flu':ab,ti OR 'influenza a(h1n1)':ab,ti OR 'swine influenza':ab,ti) AND ('attitude'/exp OR 'access to information'/exp OR 'awareness' OR 'access to information' OR 'risk perception' OR 'perceived risk' OR 'efficacy' OR 'mass communication'/exp OR 'mass communication' OR 'mass medium'/exp OR 'mass medium' OR 'mass media':ab,ti OR communicat*:ab,ti OR 'information':ti OR 'knowledge':ti) NOT ('therapeutic uses':ab,ti OR 'simulation'/exp OR 'hospital'/exp OR 'hospitalization'/exp OR 'laboratory test'/exp OR 'disease registry'/exp OR 'reproductive':ab,ti OR 'records':ti OR 'tobacco':ab,ti OR ethic*:ab,ti OR 'veterinary medicine'/exp OR 'mental disease'/exp OR 'dental':ab,ti OR 'stroke':ab,ti OR 'law':ti OR 'legal':ti OR 'hiv':ti OR 'aids':ab,ti OR 'diabetes':ab,ti OR 'surveillance':ti OR 'randomized trials':ab,ti OR 'asthma':ti OR 'drug':ti OR 'regulations':ti OR pharma*:ti OR zoonosis*:ti OR [medline]/lim) AND ([chinese]/lim OR [english]/lim OR [french]/lim OR [italian]/lim OR [portuguese]/lim OR [spanish]/lim) AND [1-1-2009]/sd NOT [2-8-2012]/sd AND [humans]/lim AND [embase]/lim AND [2009–2012]/py	15	6	3	6
	*Filters: Publication date from 2009/01/01 to 2012/08/01; Humans; English; French; Chinese; Italian; Spanish; Portuguese*				
**PsycINFO**	('((DE "Swine Influenza" OR h1n1 OR "swine flu" OR "swine influenza") AND (DE ("Communication" OR "Communication Theory" OR "Communications Media" OR "Audiovisual Communications Media" OR "Mass Media" OR "Multimedia" OR "Social Media" OR "Telecommunications Media" OR "Information" OR "Health Promotion" OR "Health Knowledge" OR "Knowledge Level") OR communication* OR media OR Knowledge OR information OR awareness))) NOT AB workers NOT AB hospital* NOT clinic*	79	58	4	17
	Limiters - Published Date: 20090101–20120831; Publication Status: fully published; Publication Type: All Journals, Peer Reviewed Journal, Peer-Reviewed Status-Unknown; Language: Chinese, English, French, Italian, Portuguese, Spanish; Document Type: Journal Article; Exclude Dissertations				
**Education Resources Information Center (ERIC)**	((DE "Swine Influenza" OR h1n1 OR "swine flu" OR "swine influenza") AND (DE ("Communication" OR "Communication Theory" OR "Communications Media" OR "Audiovisual Communications Media" OR "Mass Media" OR "Multimedia" OR "Social Media" OR "Telecommunications Media" OR "Information" OR "Health Promotion" OR "Health Knowledge" OR "Knowledge Level") OR communication* OR media OR Knowledge OR information OR awareness)) NOT databases NOT clinic	7	6	1	0
	*Limiters - Peer Reviewed; Journal or Document: Journal Articles (EJ); Language: (English OR Chinese OR French OR Italian OR Portuguese OR Spanish)*				
**Communication Abstracts**	((DE "Swine Influenza" OR h1n1 OR "swine flu" OR "swine influenza") AND (DE ("Communication" OR "Communication Theory" OR "Communications Media" OR "Audiovisual Communications Media" OR "Mass Media" OR "Multimedia" OR "Social Media" OR "Telecommunications Media" OR "Information" OR "Health Promotion" OR "Health Knowledge" OR "Knowledge Level") OR communication* OR media OR Knowledge OR information OR awareness))	16	7	3	6
	*Limiters - Scholarly (Peer Reviewed) Journals; Publication Date: 20090101–20120831; Publication Type: Academic Journal, Review; Document Type: Article*				
**Sociological abstracts**	((DE "Swine Influenza" OR h1n1 OR "swine flu" OR "swine influenza") AND (DE ("Communication" OR "Communication Theory" OR "Communications Media" OR "Audiovisual Communications Media" OR "Mass Media" OR "Multimedia" OR "Social Media" OR "Telecommunications Media" OR "Information" OR "Health Promotion" OR "Health Knowledge" OR "Knowledge Level") OR communication* OR media OR Knowledge OR information OR awareness))	10	6	3	1
	*Limiters - Peer reviewed; Source type: Scholarly Journals Additional limits - Date: From January 01 2009 to August 01 2012;*				
**Applied Social Sciences Index and Abstracts (ASSIA)**	ab(("Swine Influenza" OR h1n1 OR "swine flu" OR "swine influenza")) AND ab((DE ("Communication" OR "Communication Theory" OR "Communications Media" OR "Audiovisual Communications Media" OR "Mass Media" OR "Multimedia" OR "Social Media" OR "Telecommunications Media" OR "Information" OR "Health Promotion" OR "Health Knowledge" OR "Knowledge Level") OR communication* OR media OR Knowledge OR information OR awareness)) NOT pub((test* OR reporting OR surveillance OR nurses OR nursing))	40	25	6	9
	*Date: From January 01 2009 to August 01 2012; Language: All; Source type: Scholarly Journals*				
**Web of science**	TS = (h1n1 OR "swine flu" OR "swine influenza")	248	219	9	20
	AND				
	TS = ("communication*" OR "media" OR "knowledge" OR "information" OR "awareness")				
	NOT				
	TI = (“therapeutic use*” OR “reproductive” OR “records as topic” OR “exercise*” OR “modeling” OR “modelling” OR “tobacco” OR “ethic*” OR “veterinary medicine” OR “insurance” OR “disorders” OR "contracept*" OR "emergency medicine" OR clinical* OR “dental” OR “stroke” OR “law” OR “legal” OR “pharmacist*” OR “pharmaceutical*” OR “HIV” OR “surveillance” OR "emergency department" OR “nurse*” OR “AIDS” OR “provider*” OR “worker*” OR "departments" OR "sales")				
	NOT				
	TS = (“reproductive” OR “records as topic” OR “exercise*” OR “modeling” OR “modelling” OR “tobacco” OR “ethic*” OR “veterinary medicine” OR “insurance” OR "contracept*" OR "emergency medicine" OR “dental” OR “stroke” OR “law” OR “legal” OR “pharmacist*” OR “HIV” OR “surveillance” OR "emergency department" OR “AIDS” OR "sales")				
	Filters:				
	**LANGUAGE:***(English OR Chinese OR French OR Italian OR Portuguese OR Spanish) AND*				
	**Timespan** *= 2009-2012*				
	*Refined by: DOCUMENT TYPES = (ARTICLE) AND WEB OF SCIENCE CATEGORIES = (INFECTIOUS DISEASES OR EMERGENCY MEDICINE OR SOCIAL SCIENCES BIOMEDICAL OR SOCIAL SCIENCES INTERDISCIPLINARY OR HEALTH CARE SCIENCES SERVICES OR EDUCATION EDUCATIONAL RESEARCH OR NURSING OR PSYCHOLOGY MULTIDISCIPLINARY OR HEALTH POLICY SERVICES OR PSYCHOLOGY SOCIAL OR COMMUNICATION OR SOCIAL ISSUES OR NURSING SCI OR SOCIOLOGY)*				
**Total**		**770**	**475**	**32**	**263**

Articles were excluded if: 1) published in any other language other than the six stated above; 2) focusing solely on inter-agency communication or communication among health professionals; 3) focusing on development on telecommunication strategy such as telecommunication technology; 4) not pertaining specifically to the 2009 H1N1 pandemic; or 5) focusing on public health surveillance and epidemiologic investigations. The PRISMA guidelines were followed to conduct the review when appropriate given the non-clinical nature of the subject matter [15].

### Data extraction and analysis

Two reviewers independently read and coded the articles included for final review.

**Figure 1:**
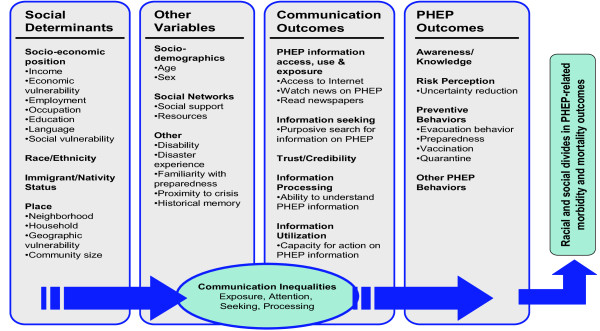
Structural Influence Model (SIM) of communication inequalities.

To characterize the articles the following criteria were used: country of origin of the first author, methodological approach (i.e. empirical studies and information environment analysis – a systematic analysis of news content on H1N1 on different information channels), study design (e.g. case study, cross sectional, experimental), data gathering methods (e.g. survey, interview, focus group) and when focused on a population study: sampling methodology (e.g., cluster, convenience, census-based). Subsequently, data were extracted from the articles using the Structural Influence Model (SIM) of PHEP Communications (Figure 1) [11,16] developed by Viswanath K. et al. This model is a heuristic framework developed to link social determinants with PHEP outcomes through PHEP communications. The articles were reviewed and coded searching for evidence of the potential association between *social determinants, attitudes and beliefs, and communication and PHEP outcomes* as outlined in the model*.* Coding agreement between the two reviewers was 90%. Disagreements were resolved through discussion between the reviewers. Descriptive statistics were performed using STATA College Station, Texas, release 12 to identify how frequently specific variables proposed in the SIM had been investigated in the empirical studies.

## Results

### Characterization of the literature

**Figure 2:**
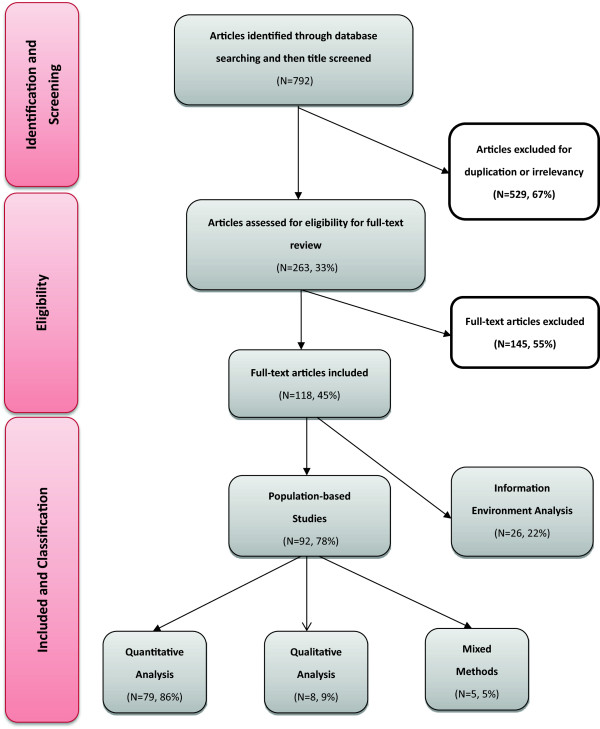
Distribution of articles by study characteristics.

We started the review with a comprehensive search of all major health and social science databases, followed by initial title scanning for duplicates and topic relevancy, which yielded a total of 263 articles eligible for full-text review. Then, we conducted a full-text review of articles screening for non-empirical studies such as working papers, editorials or letters to editors, and literature reviews, which did not meet the inclusion criteria for this review. As a result, a total of 145 articles were excluded, leaving 118 empirical studies for final review. (See Figure 2 for distribution of articles by study characteristics and Additional file 1 for full reference list).

The manuscripts were written by authors from twenty-five different countries. The country most represented in the literature was the United States (37% of the articles), followed by China (11%, mostly based in Hong Kong) and the United Kingdom (10%). Population–based studies made up 78% (N = 92) of the identified empirical studies; among them, 86% (N = 79) used a quantitative approach, 9% (N = 8) a qualitative approach and 5% (N = 5) mixed methods. The remainder of the selected papers (22%, N = 26) employed methods focused on the analysis of the “information environment”, consisting of content analysis of news coverage and examination of the style and volume of news reporting. Almost all population-based studies used cross-sectional design (91%, N = 84) and two used longitudinal design (2%). Five studies employed experimental designs. In terms of data gathering methods, surveys were used in 82% of the population–based studies, 12% used in-depth interviews and 7% used focus groups. Sometimes interviews and focus groups were conducted as complementary research methods to cross-sectional surveys. More than half of the population–based studies (55%) derived their data from a random sample of the population and 40% from a convenience sample. Few studies (<5%) solely relied on cluster sampling or sampling from an entire segment of the population.

### Communication theories

Only six of the empirical studies based their investigations of population behaviors on existing theoretical frameworks. For example, Kumar et al. used the *Social Ecological Model* as a framework while identifying determinants of 2009 H1N1 influenza vaccination acceptance among American adults [17]. Liao interviewed Hong Kong residents and used the *Structural Equation Modelling* to examine associations between the theoretical determinants (e.g. trust in information, self-efficacy, and perceived susceptibility, as well as worry) and prevention behaviors such as hand hygiene and social distancing [18]. Furthermore, Prati et al. worked to further develop an existing model, *Social-Cognitive Model,* and examined the risk perception and individual response to pandemic influenza H1N1 among the Italian population [19]. Understanding human behaviors, and/or at least behavior intentions during a pandemic influenza, has been the focus of many studies. Some drew upon existing psychology theories about behavior intentions such as *Theory of Reasoned Action*, *Theory of Planned Behavior* and the extended *Theory of Planned Behavior,* and *Integrated Behavioral Model*, to understand their study populations’ decisions about H1N1 vaccine uptake [20,21]. Furthermore, others like Li et al., in contrast to theories on the traditional perspectives of planned behavior and the maximizing and satisfying strategies, claimed that human behaviors, especially preventive health behaviors, are not only purely motivated by careful analysis of perceived costs and benefits of actions, but also by individual factors as well as social and situational constraints such as emotions, habits, and social influences [22].

#### Population-based studies

##### Sociodemographic variables

Our analytical approach started by listing the socio-demographic variables gathered by scientists when describing the study populations exposed to public H1N1 messages. Age and gender were used in 86% of the studies, and race and ethnicity were taken into consideration in 36% of the studies. Information on socioeconomic position (SEP) was reported in 73% of the empirical studies by the use of variables such as education, income and employment, either solely or in combination. Education and income/wealth were the most commonly used variables used as indicators of SEP [7]. Number(s) of children in the household was one of the most studied household characteristics (37%) frequently looked at during the 2009/2010 H1N1 pandemic. About one-third of the researchers were also interested in the area of residence of the population being investigated (i.e., urban versus rural, 27%).

##### Interpersonal and psychosocial factors

During the response to the H1N1 pandemic, population’s attitudes were extensively researched (by 46% of the studies) as an important predictor of people’s preventive and health behaviors. These attitudes were measured by pre-existing beliefs about pandemic influenza such as social stigma and discrimination against one or more particular social sub-group (s), trust in government’s handling of the emergency, and/or fairness of treatment of all social groups. Other interpersonal and psychosocial factors that played a major role in the public’s response to the pandemic were: experience with pandemic or seasonal influenza prior to H1N1 (33%), self-efficacy (32%) - defined as the level of confidence in one’s ability to undertake a recommended preventive behavior, and general health status (25%).

##### Communications and preparedness outcomes

Scientists have studied populations’ sources of information regarding the H1N1 pandemic (50%), and the perceived trust and credibility in the information received (32%). Other commonly researched public health preparedness communication behaviors were information utilization (12%) and information-seeking (11%). One thing worth noting is that communication behaviors were frequently examined as important mediating factors of population’s preparedness outcomes. The most frequently researched preparedness outcomes were: preventive behaviors such as hygiene and social distancing practices in 70% of the studies (N = 64), risk perceptions such as likelihood of getting infected, severity of the disease, and perceived susceptibility and/or vulnerability to the disease (70%, N = 64), levels of knowledge and awareness about the pandemic (53%, N = 49), and emotional responses such as fear and worry (47%, N = 43). Factors influencing the H1N1 vaccination acceptance rate (26%, N = 24) were also frequently investigated. Other communication and preparedness outcomes examined by the literature are described in Table 2.

**Table 2:** Frequency of studies addressing communication and preparedness outcomes

**Communication outcomes**	**Definition**	**Frequency (%)**	**Examples**
Information sources and exposure	The incidental exposure of information of a public health threat, which is not actively looked for by the audience, but obtained through daily routine or from the surrounding.	(46) 50%	Information about government’s social distancing recommendations learned from routine television watching.
Information seeking behaviors	The actions people take proactively to search for information about public health threats for self-protection and survival.	(10) 11%	Browsed website and/or call doctors to get info about vaccine against H1N1.
Trust and credibility	Trust and credibility in the information sources, quality of the information received, fairness of treatment, or government’s ability to respond to a public health emergency.	(29) 32%	Trust in commercial television or health department as information source about H1N1 vaccines.
Information processing	Ability to understand information about public health threats for self-protection and survival.	(3) 3%	Some subgroups in society were more vulnerable during pandemics because they had difficulty in understanding preventive measures.
Information utilization	Ability and/or willingness to use information obtained to prepare for and respond to public health threats.	(11) 12%	Compliance with hygienic practices during pandemics.
**Preparedness outcomes**	**Definition**	**Frequency (%)**	**Examples**
Knowledge/awareness	Knowledge about specific threats and preventive behaviors.	49 (53%)	Individuals with knowledge of a particular mode of transmission for H1N1.
Risk perception	Subjective judgment about the characteristics and severity of personal or societal risk.	64 (70%)	The risk of being infected with the H1N1 virus.
Preventive behaviors	Any activity undertaken by individuals to prevent a disease or limit contagion to other people.	64 (70%)	Compliance with the hygienic practices, immunization practices.
Healthcare behaviors	Any activity undertaken by individuals to seek medical attention after they encounter the threat.	24 (26%)	Seeking health care, compliance with recommended medications (i.e. antivirals).
Emotional response	Emotional reactions that occur as a response to a real risk or potential threat to health or environment.	43 (47%)	Fear, worry, anxiety, hopelessness or anger.

### Social determinants of communication inequalities during H1N1

#### Determinants of knowledge, attitude and beliefs

**Figure 3:**
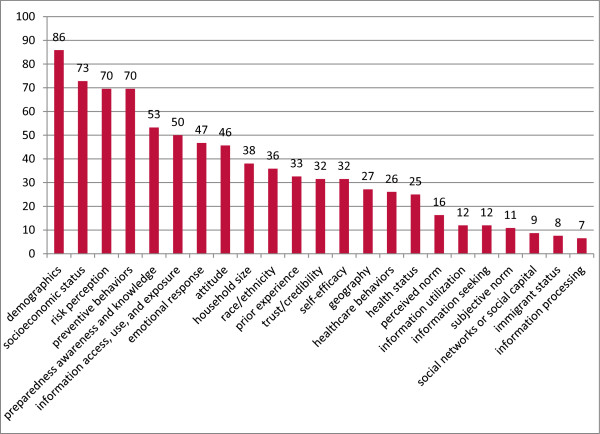
Proportions of studied determinants of communication inequalities during the H1N1 pandemic in population-based studies.

Most population-based studies (82%, N = 75) looked at the association between specific population characteristics (i.e. sociodemographic as well as interpersonal and psychosocial factors) and H1N1-related communication and/or preparedness outcomes. See Figure 3. Our data show that older age, household income, level of education, and homeownership were positively associated with greater knowledge about H1N1. Studies have shown exposure to media or public health-focused advertising campaigns had a positive impact on not only increasing people’s levels of H1N1-related knowledge, but also their adoption of health behaviors [6,23-29]. A successful example of advertising campaigns was the official public health advertising campaign launched in the U.K., which was accompanied by commercial advertisements of tissues, hand sanitizers, and other related products that regularly repeated the official hygiene messages (i.e. “Catch it, Bin it, Kill it”), that successfully increased the perceived efficacy and adoption of recommended behaviors [26]. Being worried about the disease was identified as an important predictor of compliance with recommended preventive behaviors, and to be associated with the volume of media attention and media reporting on the number of H1N1 cases [18,19,24,26,30-34]. Furthermore, many studies have presented evidence linking H1N1-related knowledge to people’s attitudes (which could be either positive or negative evaluations of particular behaviors or events) such as approval of the governments’ response to the pandemic [20,35-37]. Knowledge and attitudes about the H1N1 pandemic had an impact on people’s adoption of preventive measures [19,24,26,27,29,35,38-43]. Knowing how the H1N1 virus was transmitted and having a good understanding of the symptoms of infection were positively associated with perceived risk and perceived susceptibility to infection, and belief in the effectiveness of recommended behaviors, which affected response efficacy [26,27,35,38-44]. Compliance with social distancing and hygiene measures (i.e. hand washing), was found to be greater in those of older age, in parents of children aged 18 or less, in women compared to men, and in people with higher SEP [18,35,38,39,41,42,45-48].

Attitudinal determinants leading to greater compliance with recommended behaviors included levels of worry about self or family members at risk of catching the flu, perceiving the disease as potentially severe and belief of being susceptible to the infection [18,35,38]. Trust in the source of recommendation was associated with compliance with non-pharmaceutical measures [18,19,49-52]. Indeed, studies show that trust and credibility are the essential elements in all risk communication strategies and significantly affect people’s choice of information sources and attitudes towards the message received [18,19,44,49]. People perceived their social networks including friends, family, and physicians, as well as communities (e.g. work place and church) and health agencies, as trustworthy sources of information [21,29,30,44,53].

### Determinants of vaccine uptake

Vaccine uptake was positively associated with many of the same factors as knowledge and attitudes, namely older age, social capital, worry, media exposure, and information-seeking behaviors, as well as perceived severity and susceptibility to infection [17,19,21,24-26,30,53-56]. Those who felt official authorities had openly provided the public with clear and honest information about pandemic influenza vaccination believed to be sufficiently informed and were more likely to get immunized [19,21,22,25,44,51,52,55,57]. It is also worth noting that people with different educational levels rely on different sources of information with television being the most popular source for people with lower education and the internet for those with higher education [57]. Trust in public officials and having a doctor recommending the vaccine have been reported to have a positive impact on people’s acceptance of vaccination [21,25,26,51,52,55,57-60]. Having a history of acceptance of the seasonal flu vaccine played an important role in vaccine uptake [8,36,48,51,52,54,59-61]. Perceiving the vaccination as safe and effective was shown to be positively associated with vaccine uptake, while being concerned about potential side effects was negatively associated with such behavior [8,22,51,56,58-60]. Knowing someone affected by the H1N1 flu also motivated people to accept the vaccine; however, the cost of the vaccines did discourage some from getting it [25,62-64]. Race and ethnicity also played a role in vaccine uptake with being African American as a potential predictor of lower compliance with this immunization practice [20,21,58,65]. In particular, studies found spread of rumors of vaccine unsafety in the African American communities that countered the health departments’ recommendations and decreased vaccine demand; collaboration with community based organizations such as churches in the dissemination of the messages has been recommended as a way to overcome issues of trust from such communities [21,58]. On the other hand, in contrast to the usually low acceptance rate of seasonal flu vaccine, the Hispanic population in the United States was found to be more likely to get the H1N1 vaccine [17,58,66,67]. The fact that the original H1N1 cases were first identified in Mexico likely caused this segment of the population to be aware of the risk of infection and have a different pandemic experience compared to other ethnic groups. Unfortunately this group also experienced social stigmatization because of the origin of the pandemic [68,69].

#### Information environment analysis

In recent years, there has been increasing attention to the analysis of the information environment during large-scale emergencies. In our review, 22% of empirical studies employed information environment analysis techniques during the recent H1N1 pandemic. Researchers assessed the content, timeliness, or volume of information available used to study the quality (such as accuracy, accessibility, and language style) and/or quantity of news media, press releases, online comments in response to news articles or social media, and H1N1-related information available on government or school websites. Some results were then compared to existing H1N1-related behavioral data, such as access to healthcare or vaccine uptake, in order to estimate the impact of media coverage on people’s attitudes and behaviors [31,32,70,71]. *Blogs* and *wiki visits* were examined to understand public attitudes and information-seeking behaviors. Somewhat surprisingly, popular social media tools such as *Twitter* and *Facebook* and their impact on PHEP communication outcomes were relatively rarely evaluated [21,29,49,72-75]. Furthermore, it is also important to emphasize that, although *Facebook* and *Twitter* have been recognized as useful tools to raise awareness and promote intervention measures [21,49,72-75], the traditional forms of mass media, as well as communications through pre-established social networks, remained the primary source of information [21,29,30,44,53].

## Discussion

The H1N1 pandemic has challenged public health officials around the globe in developing effective communication strategies in absence of clear information on the severity and transmissibility of the virus. Media “hype” and uncertainty of information have caused panic at the beginning of the pandemic. However, a few months into the pandemic when vaccine became ready for distribution, the severity of the disease was deemed to be mild and a lack of compliance with recommended preventive behaviors prevailed. Given the complexity of the scenario, the H1N1 pandemic has prompted researchers around the globe to study populations’ reactions to such events. This literature review provides an analysis and synthesis of data derived from 118 empirical studies focused on the H1N1 pandemic, investigating differences across various segments of the population in communication and preparedness outcomes. This review is important for the public health practitioner because communication strategies can be designed to address the impact of social factors, as well as people’s attitudes and beliefs on communication outcomes and preparedness behaviors only when evidence on the role of specific factors exists. The literature being examined demonstrates the existence of differences across various segments of the population on information exposure during the H1N1 pandemic as well as their reactions and behaviors. The literature does not examine the effectiveness of specific communication strategies, but few recommendations may be derived and suggested for public health practitioners engaged in the development of communication strategies. This review suggests that to reduce communication inequalities during a large scale emergency, such as a pandemic, public health officials should focus their communication efforts on the young, the less educated and the indigent because there is evidence that these are the people at risk of not knowing about the threat, perceiving the threat to be of low risk and ultimately being less likely to follow recommended behaviors. Exposure to various communication channels varies by educational level with the less educated being exposed to television rather than newspapers or other means of communication [57]. This result suggests the need to build and sustain, over the course of an emergency, collaboration between public health agencies and media in order to disseminate information in a timely and accurate manner, thereby avoiding hypes and lows in the flow of information and developing a strategy that counterbalances the inevitable spread of rumors on the safety of recommended practices (i.e. immunization) and on the overall impact of the threat. An honest reporting of what the threat looks like, through a presentation of known and unknown factors, seems to have a better impact on people’s knowledge, attitudes and beliefs, including trust in the way the government is handling the emergency. Consequently, there is some evidence that better knowledge and trust are likely to be associated with the adoption of recommended behaviors (i.e. immunization practices). Social networks and ties to the community are also drivers of better knowledge and compliance with preventive measures; these results suggest that non-traditional channels of communication (i.e. partnership with community leaders or organizations) should be used to reach out to the most vulnerable and those in need of a better understanding of the risks and actions needed to be able to protect themselves. The fact that during the H1N1 pandemic, people with a higher educational level were better informed about the risks is not a surprise but suggests that public health communication messages are still delivered at a literacy level that does not meet the needs of the less educated [19,76]. Some limitations are to be highlighted when interpreting these results. Most empirical studies included in the review had a cross-sectional study design and, therefore, conclusions cannot be drawn on the causal relationship between some determinants and specific communication and preparedness outcomes. Longitudinal studies or experiments are required to provide stronger evidence for such relationships. The selected studies were conducted at different points in time during the H1N1 pandemic and across various countries making the interpretation of aggregated results difficult. For example, differences in the H1N1 vaccination policies imposed across the globe in terms of decisions about the target populations and timing of influenza vaccination, prioritization of vaccine distribution, and policy of non-compliance might explain some of the variations in the findings among the studies. Also, racial disparities bear different interpretations among different countries such as the United States, Malaysia, and China. However, the recommendations provided above seem to be valid across the globe with a need for public health practitioners to develop messages targeted to specific groups to reduce disparities in the adoption of preventive behaviors.

## Conclusion

Consistently across studies, a list of potential predictors of behavioral compliance to preventive recommendations during a pandemic were identified, including socio-demographic characteristics (e.g. age and race and ethnicity), attitudinal factors (e.g. perceived severity) and communication determinants (e.g. media exposure, information seeking behaviors and levels of knowledge about the threat). Understanding these factors can help us tailor public health communication messages to the target audience according to their social-demographic characteristics by calibrating the messaging format and optimizing channels of communication. When addressed in interventions, it can close the gap of communication inequality and increase the effectiveness of the preparedness and response to influenza pandemics. Most importantly, data showed it will be critical to work with community leaders, physicians, and communication specialists, as well as mass media, to improve the reach, accuracy, and timeliness of public health messages. This will ensure the highest rates of compliance with recommended protection measures and reduce communication inequalities during future emergencies.

## Abbreviations

PHEP: Public health emergency preparedness; SIM: Structural influence model of PHEP communications; SEP: Socioeconomic positions.

## Competing interests

The authors declare that they have no competing interests.

## Authors’ contributions

LL collected the data, coded and analyzed, and interpreted the data. LL drafted the manuscript and helped complete the PRISMA checklist. ES designed the study and helped code, analyze and interpret the data. ES helped draft, revise and review the manuscript. FA helped code, analyze and interpret the data. FA helped draft, revise and review the manuscript as well as completed the PRISMA checklist. KV designed the study, helped draft the manuscript, and reviewed all drafts of the manuscript. All authors agree with the manuscript results and conclusions. All authors read and approved the final manuscript.

## Pre-publication history

The pre-publication history for this paper can be accessed here:

http://www.biomedcentral.com/1471-2458/14/484/prepub

## Supplementary Material

Additional file 1 **Population-based studies.** Information environment analysis. Click here for file
