# Supplementary material for: What have we learned about communication inequalities during the H1N1 pandemic: a systematic review of the literature
Source: BMC Public Health. 2014 May 21;14:484. doi: 10.1186/1471-2458-14-484 (PMC4048599; doi:10.1186/1471-2458-14-484)
Supplement: Additional file 1 — Population-based studies. Information environment analysis. [file 1471-2458-14-484-S1.doc]

**Additional file**

***Population-Based Studies***

1. Aburto NJ, Pevzner E, Lopez-Ridaura R, Rojas R, Lopez-Gatell H, Lazcano E, Hernandez-Avila M, Harrington TA: **Knowledge and adoption of community mitigation efforts in Mexico during the 2009 H1N1 pandemic**. *American Journal of Preventive Medicine* 2010, **39**(5):395–402.

2. Alsaif A, Al-Sagair O, Albarrak A, Ginawi I, Hussein T, Sweelam M: **Hail community acceptance of a/H1N1 vaccine**. *J Med Sci* 2010, **10**(6):162–168.

3. Asgary A: **Assessing households' willingness to pay for an immediate pandemic influenza vaccination programme**. *Scandinavian Journal of Public Health* 2012, **40**(5):412–417.

4. Austin EW, Pinkleton BE, Austin BW, Van de Vord R: **The relationships of information efficacy and media literacy skills to knowledge and self-efficacy for health-related decision making**. *Journal of American college health : J of ACH* 2012, **60**(8):548–554.

5. Balkhy HH, Abolfotouh MA, Al-Hathlool RH, Al-Jumah MA: **Awareness, attitudes, and practices related to the swine influenza pandemic among the Saudi public**. *BMC infectious diseases* 2010, **10**(Journal Article):42-2334-2310-2342.

6. Bangerter A, Krings F, Mouton A, Gilles I, Green EG, Clemence A: **Longitudinal investigation of public trust in institutions relative to the 2009 H1N1 pandemic in Switzerland**. *PloS one* 2012, **7**(11):e49806.

7. Boyd CA, Gazmararian JA, Thompson WW: **Knowledge, Attitudes, and Behaviors of Low-Income Women Considered High Priority for Receiving the Novel Influenza A (H1N1) Vaccine**. *Maternal and child health journal* 2012(Journal Article).

8. Caress AL, Duxbury P, Woodcock A, Luker KA, Ward D, Campbell M, Austin L: **Exploring the needs, concerns and behaviours of people with existing respiratory conditions in relation to the H1N1 'swine influenza' pandemic: a multicentre survey and qualitative study**. *Health technology assessment (Winchester, England)* 2010, **14**(34):1–108.

9. Cassady D, Castaneda X, Ruelas MR, Vostrejs MM, Andrews T, Osorio L: **Pandemics and vaccines: perceptions, reactions, and lessons learned from hard-to-reach Latinos and the H1N1 campaign**. *Journal of health care for the poor and underserved* 2012, **23**(3):1106–1122.

10. Catellier J, Yang Z: **Trust and affect: how do they impact risk information seeking in a health context?** *Journal of Risk Research* 2012, **15**(8):897–911.

11. Chanel O, Luchini S, Massoni S, Vergnaud JC: **Impact of information on intentions to vaccinate in a potential epidemic: Swine-origin Influenza A (H1N1)**. *Social science & medicine (1982)* 2011, **72**(2):142–148.

12. Charania NA, Tsuji LJ: **Government bodies and their influence on the 2009 H1N1 health sector pandemic response in remote and isolated First Nation communities of sub-Arctic Ontario, Canada**. *Rural and remote health* 2011, **11**(3):1781.

13. Chien YH: **Message framing and color combination in the perception of medical information**. *Psychological reports* 2011, **108**(2):667–672.

14. Chien YH: **Message framing and color combination in the perception of medical information**. *Psychological reports* 2011, **108**(2):667–672.

15. Cowling BJ, Ng DM, Ip DK, Liao Q, Lam WW, Wu JT, Lau JT, Griffiths SM, Fielding R: **Community psychological and behavioral responses through the first wave of the 2009 influenza A(H1N1) pandemic in Hong Kong**. *The Journal of infectious diseases* 2010, **202**(6):867–876.

16. Davila ME, Mujica MJ, Bullones X, Marrufo MP, Daza D: **Level of knowledge regarding influenza A (H1N1) 2009**. *Revista de salud publica (Bogota, Colombia)* 2010, **12**(5):790–797.

17. Davis M, Stephenson N, Flowers P: **Compliant, complacent or panicked? Investigating the problematisation of the Australian general public in pandemic influenza control**. *Social science & medicine (1982)* 2011, **72**(6):912–918.

18. Eastwood K, Durrheim DN, Jones A, Butler M: **Acceptance of pandemic (H1N1) 2009 influenza vaccination by the Australian public**. *The Medical journal of Australia* 2010, **192**(1):33–36.

19. Ferrante G, Baldissera S, Moghadam PF, Carrozzi G, Trinito MO, Salmaso S: **Surveillance of perceptions, knowledge, attitudes and behaviors of the Italian adult population (18–69 years) during the 2009–2010 A/H1N1 influenza pandemic**. *European journal of epidemiology* 2011, **26**(3):211–219.

20. Freiman AJ, Montgomery JP, Green JJ, Thomas DL, Kleiner AM, Boulton ML: **Did H1N1 influenza prevention messages reach the vulnerable population along the Mississippi Gulf Coast?** *Journal of public health management and practice : JPHMP* 2011, **17**(1):52–58.

21. Frew PM, Painter JE, Hixson B, Kulb C, Moore K, del Rio C, Esteves-Jaramillo A, Omer SB: **Factors mediating seasonal and influenza A (H1N1) vaccine acceptance among ethnically diverse populations in the urban south**. *Vaccine* 2012, **30**(28):4200–4208.

22. Galarce EM, Minsky S, Viswanath K: **Socioeconomic status, demographics, beliefs and A(H1N1) vaccine uptake in the United States**. *Vaccine* 2011, **29**(32):5284–5289.

23. Gaygisiz U, Gaygisiz E, Ozkan T, Lajunen T: **Individual differences in behavioral reactions to H1N1 during a later stage of the epidemic**. *Journal of infection and public health* 2012, **5**(1):9–21.

24. Gidengil CA, Parker AM, Zikmund-Fisher BJ: **Trends in risk perceptions and vaccination intentions: a longitudinal study of the first year of the H1N1 pandemic**. *American journal of public health* 2012, **102**(4):672–679.

25. Goodwin R, Gaines SO, Jr., Myers L, Neto F: **Initial psychological responses to swine flu**. *International journal of behavioral medicine* 2011, **18**(2):88–92.

26. Gray L, MacDonald C, Mackie B, Paton D, Johnston D, Baker MG: **Community responses to communication campaigns for influenza A (H1N1): a focus group study**. *BMC public health* 2012, **12**(Journal Article):205-2458-2412-2205.

27. Griffiths SM, Wong AH, Kim JH, Yung TK, Lau JT: **Influence of country of study on student responsiveness to the H1N1 pandemic**. *Public health* 2010, **124**(8):460–466.

28. Hilton S, Smith E: **Public views of the UK media and government reaction to the 2009 swine flu pandemic**. *BMC public health* 2010, **10**(Journal Article):697-2458-2410-2697.

29. Hilyard KM, Freimuth VS, Musa D, Kumar S, Quinn SC: **The vagaries of public support for government actions in case of a pandemic**. *Health affairs (Project Hope)* 2010, **29**(12):2294–2301.

30. Horney JA, Moore Z, Davis M, MacDonald PD: **Intent to receive pandemic influenza A (H1N1) vaccine, compliance with social distancing and sources of information in NC, 2009**. *PloS one* 2010, **5**(6):e11226.

31. Hutchinson AF, Thompson MA, Clark L, Irving LB: **Communicating information regarding human H1N1-09 virus to high-risk consumers: knowledge and understanding of COPD patients in Melbourne, Australia**. *Collegian (Royal College of Nursing, Australia)* 2010, **17**(4):199–205.

32. Ibuka Y, Chapman GB, Meyers LA, Li M, Galvani AP: **The dynamics of risk perceptions and precautionary behavior in response to 2009 (H1N1) pandemic influenza**. *BMC infectious diseases* 2010, **10**(Journal Article):296-2334-2310-2296.

33. Jehn M, Kim Y, Bradley B, Lant T: **Community knowledge, risk perception, and preparedness for the 2009 influenza A/H1N1 pandemic**. *Journal of public health management and practice : JPHMP* 2011, **17**(5):431–438.

34. Jhummon-Mahadnac ND, Knott J, Marshall C: **A cross-sectional study of pandemic influenza health literacy and the effect of a public health campaign**. *BMC research notes* 2012, **5**(Journal Article):377-0500-0505-0377.

35. Jung M, Lin L, Viswanath K: **Associations between health communication behaviors, neighborhood social capital, vaccine knowledge, and parents' H1N1 vaccination of their children**. *Vaccine* 2013, **31**(42):4860–4866.

36. Kamal NN, Seedhom AE: **Knowledge, attitude and practice of El-Minia university students towards pandemic H1N1, Egypt, 2009**. *Journal of Public Health* 2011, **19**(6):505–510.

37. Kamate SK, Agrawal A, Chaudhary H, Singh K, Mishra P, Asawa K: **Public knowledge, attitude and behavioural changes in an Indian population during the Influenza A (H1N1) outbreak**. *Journal of infection in developing countries* 2009, **4**(1):7–14.

38. Kanadiya MK, Sallar AM: **Preventive behaviors, beliefs, and anxieties in relation to the swine flu outbreak among college students aged 18–24 years**. *Journal of Public Health* 2011, **19**(2):139–145.

39. Kavanagh AM, Bentley RJ, Mason KE, McVernon J, Petrony S, Fielding J, LaMontagne AD, Studdert DM: **Sources, perceived usefulness and understanding of information disseminated to families who entered home quarantine during the H1N1 pandemic in Victoria, Australia: a cross-sectional study**. *BMC infectious diseases* 2011, **11**(Journal Article):2-2334-2311-2332.

40. Kiviniemi MT, Ram PK, Kozlowski LT, Smith KM: **Perceptions of and willingness to engage in public health precautions to prevent 2009 H1N1 influenza transmission**. *BMC public health* 2011, **11**(Journal Article):152-2458-2411-2152.

41. Kumar N, Sood S, Singh M, Kumar M, Makkar B: **Knowledge of swine flu among Health Care workers and General Population of Haryana India during 2009 pandemic**. *AMJ* 2010, **3**(9):614–617.

42. Kumar S, Quinn SC, Kim KH, Musa D, Hilyard KM, Freimuth VS: **The social ecological model as a framework for determinants of 2009 H1N1 influenza vaccine uptake in the United States**. *Health education & behavior : the official publication of the Society for Public Health Education* 2012, **39**(2):229–243.

43. Lau JT, Griffiths S, Au DW, Choi KC: **Changes in knowledge, perceptions, preventive behaviours and psychological responses in the pre-community outbreak phase of the H1N1 epidemic**. *Epidemiology and infection* 2011, **139**(1):80–90.

44. Lau JT, Griffiths S, Choi KC, Tsui HY: **Widespread public misconception in the early phase of the H1N1 influenza epidemic**. *The Journal of infection* 2009, **59**(2):122–127.

45. Lau JT, Yeung NC, Choi KC, Cheng MY, Tsui HY, Griffiths S: **Acceptability of A/H1N1 vaccination during pandemic phase of influenza A/H1N1 in Hong Kong: population based cross sectional survey**. *BMJ (Clinical research ed)* 2009, **339**(Journal Article):b4164.

46. Lau JT, Yeung NC, Choi KC, Cheng MY, Tsui HY, Griffiths S: **Factors in association with acceptability of A/H1N1 vaccination during the influenza A/H1N1 pandemic phase in the Hong Kong general population**. *Vaccine* 2010, **28**(29):4632–4637.

47. Leggat PA, Brown LH, Aitken P, Speare R: **Level of concern and precaution taking among Australians regarding travel during pandemic (H1N1) 2009: results from the 2009 Queensland Social Survey**. *Journal of travel medicine* 2010, **17**(5):291–295.

48. Li M, Chapman GB, Ibuka Y, Meyers LA, Galvani A: **Who got vaccinated against H1N1 pandemic influenza? A longitudinal study in four U.S. cities**. *Psychology & health* 2012, **27**(1):101–115.

49. Liao Q, Cowling B, Lam WT, Ng MW, Fielding R: **Situational awareness and health protective responses to pandemic influenza A (H1N1) in Hong Kong: a cross-sectional study**. *PloS one* 2010, **5**(10):e13350.

50. Lin Y, Huang L, Nie S, Liu Z, Yu H, Yan W, Xu Y: **Knowledge, attitudes and practices (KAP) related to the pandemic (H1N1) 2009 among Chinese general population: a telephone survey**. *BMC infectious diseases* 2011, **11**(Journal Article):128-2334-2311-2128.

51. Mak KK, Lai CM: **Knowledge, risk perceptions, and preventive precautions among Hong Kong students during the 2009 influenza A (H1N1) pandemic**. *American Journal of Infection Control* 2012, **40**(3):273–275.

52. Maurer J, Harris KM: **Contact and communication with healthcare providers regarding influenza vaccination during the 2009–2010 H1N1 pandemic**. *Preventive medicine* 2011, **52**(6):459–464.

53. Maurer J, Uscher-Pines L, Harris KM: **Perceived seriousness of seasonal and A(H1N1) influenzas, attitudes toward vaccination, and vaccine uptake among U.S. adults: does the source of information matter?** *Preventive medicine* 2010, **51**(2):185–187.

54. McDonnell WM, Nelson DS, Schunk JE: **Should we fear "flu fear" itself? Effects of H1N1 influenza fear on ED use**. *The American journal of emergency medicine* 2012, **30**(2):275–282.

55. Miao YY, Huang JH: **Prevalence and associated psychosocial factors of increased hand hygiene practice during the influenza A/H1N1 pandemic: findings and prevention implications from a national survey in Taiwan**. *Tropical medicine & international health : TM & IH* 2012, **17**(5):604–612.

56. Milanesi R, Caregnato RC, Wachholz NI: **Pandemic Influenza A (H1N1): changing population health habits in Cachoeira do Sul, Rio Grande do Sul State, Brazil, 2010**. *Cadernos de saude publica* 2011, **27**(4):723–732.

57. Mitchell T, Dee DL, Phares CR, Lipman HB, Gould LH, Kutty P, Desai M, Guh A, Iuliano AD, Silverman P *et al.*: **Non-pharmaceutical interventions during an outbreak of 2009 pandemic influenza A (H1N1) virus infection at a large public university, April-May 2009**. *Clinical infectious diseases : an official publication of the Infectious Diseases Society of America* 2011, **52 Suppl 1**(Journal Article):S138-145.

58. Myers LB, Goodwin R: **Determinants of adults' intention to vaccinate against pandemic swine flu**. *BMC public health* 2011, **11**(1):15-2458-2411-2415.

59. Naing C, Tan RY, Soon WC, Parakh J, Sanggi SS: **Preventive behaviours towards influenza A(H1N1)pdm09 and factors associated with the intention to take influenza A(H1N1)pdm09 vaccination**. *Journal of infection and public health* 2012, **5**(6):412–419.

60. Naing C, Tan RYP: **Knowledge about the pandemic influenza A (H1N1) and willingness to accept vaccination: A cross-sectional survey**. *Journal of Public Health* 2011, **19**(6):511–516.

61. Nan X, Xie B, Madden K: **Acceptability of the H1N1 vaccine among older adults: the interplay of message framing and perceived vaccine safety and efficacy**. *Health communication* 2012, **27**(6):559–568.

62. Park JH, Cheong HK, Son DY, Kim SU, Ha CM: **Perceptions and behaviors related to hand hygiene for the prevention of H1N1 influenza transmission among Korean university students during the peak pandemic period**. *BMC infectious diseases* 2010, **10**(Journal Article):222-2334-2310-2222.

63. Plough A, Bristow B, Fielding J, Caldwell S, Khan S: **Pandemics and health equity: lessons learned from the H1N1 response in Los Angeles County**. *Journal of public health management and practice : JPHMP* 2011, **17**(1):20–27.

64. Prati G, Pietrantoni L, Zani B: **Compliance with recommendations for pandemic influenza H1N1 2009: the role of trust and personal beliefs**. *Health education research* 2011, **26**(5):761–769.

65. Prati G, Pietrantoni L, Zani B: **A social-cognitive model of pandemic influenza H1N1 risk perception and recommended behaviors in Italy**. *Risk analysis : an official publication of the Society for Risk Analysis* 2011, **31**(4):645–656.

66. Ramsey MA, Marczinski CA: **College students' perceptions of H1N1 flu risk and attitudes toward vaccination**. *Vaccine* 2011, **29**(44):7599–7601.

67. Redelings MD, Piron J, Smith LV, Chan A, Heinzerling J, Sanchez KM, Bedair D, Ponce M, Kuo T: **Knowledge, attitudes, and beliefs about seasonal influenza and H1N1 vaccinations in a low-income, public health clinic population**. *Vaccine* 2012, **30**(2):454–458.

68. Remmerswaal D, Muris P: **Children's fear reactions to the 2009 Swine Flu pandemic: the role of threat information as provided by parents**. *Journal of anxiety disorders* 2011, **25**(3):444–449.

69. Rubin GJ, Amlot R, Page L, Wessely S: **Public perceptions, anxiety, and behaviour change in relation to the swine flu outbreak: cross sectional telephone survey**. *BMJ (Clinical research ed)* 2009, **339**(Journal Article):b2651.

70. Rubin GJ, Potts HW, Michie S: **The impact of communications about swine flu (influenza A H1N1v) on public responses to the outbreak: results from 36 national telephone surveys in the UK - Study 1: The influence of the media on levels of worry in the community**. *Health technology assessment (Winchester, England)* 2010, **14**(34):183–266.

71. Rubin GJ, Potts HW, Michie S: **The impact of communications about swine flu (influenza A H1N1v) on public responses to the outbreak: results from 36 national telephone surveys in the UK - Study 2: Factors predicting likely acceptance of vaccination against¬†swine or seasonal flu**. *Health technology assessment (Winchester, England)* 2010, **14**(34):183–266.

72. Rubin GJ, Potts HW, Michie S: **The impact of communications about swine flu (influenza A H1N1v) on public responses to the outbreak: results from 36 national telephone surveys in the UK - Study 3: The effects of advertising and media coverage on behavioural change¬†during the early stages of the swine flu**. *Health technology assessment (Winchester, England)* 2010, **14**(34):183–266.

73. Sarmiento MP, Suarez O, Sanabria JA, Perez CE, Cadena Ldel P, Nino ME: **Knowledge and practices about the prevention and the control of the influenza A H1N1 in the community of Florida Blanca, Santander**. *Biomedica : revista del Instituto Nacional de Salud* 2011, **31**(1):91–99.

74. Savoia E, Testa MA, Viswanath K: **Predictors of knowledge of H1N1 infection and transmission in the U.S. population**. *BMC public health* 2012, **12**(Journal Article):328-2458-2412-2328.

75. Schwarzinger M, Flicoteaux R, Cortarenoda S, Obadia Y, Moatti JP: **Low acceptability of A/H1N1 pandemic vaccination in French adult population: did public health policy fuel public dissonance?** *PloS one* 2010, **5**(4):e10199.

76. Setbon M, Le Pape MC, Letroublon C, Caille-Brillet AL, Raude J: **The public's preventive strategies in response to the pandemic influenza A/H1N1 in France: distribution and determinants**. *Preventive medicine* 2011, **52**(2):178–181.

77. Soto Mas F, Olivarez A, Jacobson HE, Hsu CE, Miller J: **Risk communication and college students: the 2009 H1N1 pandemic influenza**. *Preventive medicine* 2011, **52**(6):473–474.

78. SteelFisher GK, Blendon RJ, Ward JR, Rapoport R, Kahn EB, Kohl KS: **Public response to the 2009 influenza A H1N1 pandemic: a polling study in five countries**. *The Lancet infectious diseases* 2012, **12**(11):845–850.

79. Suresh PS, Thejaswini V, Rajan T: **Factors associated with 2009 pandemic influenza A (H1N1) vaccination acceptance among university students from India during the post-pandemic phase**. *BMC infectious diseases* 2011, **11**(Journal Article):205-2334-2311-2205.

80. Teasdale E, Yardley L: **Understanding responses to government health recommendations: public perceptions of government advice for managing the H1N1 (swine flu) influenza pandemic**. *Patient education and counseling* 2011, **85**(3):413–418.

81. Tucker Edmonds BM, Coleman J, Armstrong K, Shea JA: **Risk perceptions, worry, or distrust: what drives pregnant women's decisions to accept the H1N1 vaccine?** *Maternal and child health journal* 2011, **15**(8):1203–1209.

82. Updegraff JA, Emanuel AS, Gallagher KM, Steinman CT: **Framing flu prevention--an experimental field test of signs promoting hand hygiene during the 2009–2010 H1N1 pandemic**. *Health psychology : official journal of the Division of Health Psychology, American Psychological Association* 2011, **30**(3):295–299.

83. Walter D, Bohmer M, Reiter S, Krause G, Wichmann O: **Risk perception and information-seeking behaviour during the 2009/10 influenza A(H1N1)pdm09 pandemic in Germany**. *Euro surveillance : bulletin Europeen sur les maladies transmissibles = European communicable disease bulletin* 2012, **17**(13):20131.

84. Wheaton MG, Abramowitz JS, Berman NC, Fabricant LE, Olatunji BO: **Psychological Predictors of Anxiety in Response to the H1N1 (Swine Flu) Pandemic**. *Cogn Ther Res* 2012, **36**(Journal Article):210–218.

85. Williams L, Regagliolo A, Rasmussen S: **Predicting psychological responses to influenza A, H1N1 ("swine flu"): the role of illness perceptions**. *Psychology, Health & Medicine* 2012, **17**(4):383–391.

86. Wong LP, Sam IC: **Factors influencing the uptake of 2009 H1N1 influenza vaccine in a multiethnic Asian population**. *Vaccine* 2010, **28**(28):4499–4505.

87. Wong LP, Sam IC: **Public sources of information and information needs for pandemic influenza A(H1N1)**. *Journal of community health* 2010, **35**(6):676–682.

88. Wong LP, Sam IC: **Temporal changes in psychobehavioral responses during the 2009 H1N1 influenza pandemic**. *Preventive medicine* 2010, **51**(1):92–93.

89. Wong LP, Sam IC: **Knowledge and attitudes in regard to pandemic influenza A(H1N1) in a multiethnic community of Malaysia**. *International journal of behavioral medicine* 2011, **18**(2):112–121.

90. Wong LP, Sam IC: **Behavioral responses to the influenza A(H1N1) outbreak in Malaysia**. *Journal of Behavioral Medicine* 2011, **34**(1):23–31.

91. Yip MP, Ong B, Painter I, Meischke H, Calhoun B, Tu SP: **Information-seeking behaviors and response to the H1N1 outbreak in Chinese limited-English proficient individuals living in King County, Washington**. *American journal of disaster medicine* 2009, **4**(6):353–360.

92. Zairina AR, Nooriah MS, Yunus AM: **Knowledge and practices towards influenza A (H1N1) among adults in three residential areas in Tampin Negeri Sembilan: a cross sectional survey**. *The Medical journal of Malaysia* 2011, **66**(3):207–213.

***Information Environment Analysis***

1. Bentley RA, Ormerod P: **A rapid method for assessing social versus independent interest in health issues: a case study of 'bird flu' and 'swine flu'**. *Social science & medicine (1982)* 2010, **71**(3):482–485.

2. Chang C: **News coverage of health-related issues and its impacts on perceptions: Taiwan as an example**. *Health communication* 2012, **27**(2):111–123.

3. Chew C, Eysenbach G: **Pandemics in the age of Twitter: content analysis of Tweets during the 2009 H1N1 outbreak**. *PloS one* 2010, **5**(11):e14118.

4. Ding H, Zhang J: **Social Media and participatory risk communication during the H1N1 flu epidemic: A comparative study of United States and China**. *China Media Research* 2010, **6**(4):1005.

5. Duncan B: **How the media reported the first days of the pandemic (H1N1) 2009: results of EU-wide media analysis**. *Euro surveillance : bulletin Europeen sur les maladies transmissibles = European communicable disease bulletin* 2009, **14**(30):19286.

6. Fogarty AS, Holland K, Imison M, Blood RW, Chapman S, Holding S: **Communicating uncertainty--how Australian television reported H1N1 risk in 2009: a content analysis**. *BMC public health* 2011, **11**(Journal Article):181-2458-2411-2181.

7. Gao F, Zhang M, Sadri S: **Newspapers Use More Sources Compared To Health Blogs in H1N1/Swine Flu Coverage**. *Newspaper Research Journal* 2011, **32**(2):89–96.

8. Goodall C, Sabo J, Cline R, Egbert N: **Threat, efficacy, and uncertainty in the first 5 months of national print and electronic news coverage of the H1N1 virus**. *Journal of health communication* 2012, **17**(3):338–355.

9. Henrich N, Holmes B: **What the public was saying about the H1N1 vaccine: perceptions and issues discussed in on-line comments during the 2009 H1N1 pandemic**. *PloS one* 2011, **6**(4):e18479.

10. Hilton S, Hunt K: **UK newspapers' representations of the 2009–10 outbreak of swine flu: one health scare not over-hyped by the media?** *Journal of epidemiology and community health* 2011, **65**(10):941–946.

11. Holland K, Blood RW, Imison M, Chapman S, Fogarty AS: **Risk, expert uncertainty, and Australian news media: Public and private faces of expert opinion during the 2009 swine flu pandemic**. *Journal of Risk Research* 2012, **15**(6):657–671.

12. Hortiguera H: **La sospecha end√©mica argentina. La epidemia del miedo y la producci√≥n de (des)confianza en √©pocas de crisis sanitaria**. *Estudios sobre el Mensaje Period√≠stico* 2010, **16**(Journal Article):209–234.

13. Keramarou M, Cottrell S, Evans MR, Moore C, Stiff RE, Elliott C, Thomas DR, Lyons M, Salmon RL: **Two waves of pandemic influenza A(H1N1) 2009 in Wales--the possible impact of media coverage on consultation rates, April-December 2009**. *Euro surveillance : bulletin Europeen sur les maladies transmissibles = European communicable disease bulletin* 2011, **16**(3):19772.

14. Kim S, Pinkerton T, Ganesh N: **Assessment of H1N1 questions and answers posted on the Web**. *American Journal of Infection Control* 2012, **40**(3):211–217.

15. Lagasse LP, Rimal RN, Smith KC, Storey JD, Rhoades E, Barnett DJ, Omer SB, Links J: **How accessible was information about H1N1 flu? Literacy assessments of CDC guidance documents for different audiences**. *PloS one* 2011, **6**(10):e23583.

16. Lee ST, Basnyat I: **From press release to news: mapping the framing of the 2009 H1N1 A influenza pandemic**. *Health communication* 2013, **28**(2):119–132.

17. Liu BF, Kim S: **How organizations framed the 2009 H1N1 pandemic via social and traditional media: Implications for U.S. health communicators**. *Public Relations Review* 2011, **37**(Journal Article):233–244.

18. Liuccio M, Amorese V, Miconi A, Romano V, Toscano E: **["New" virus and "old" risks. Reflections on H1N1 pandemic in the newspapers in Italy, France and UK]**. *La Clinica terapeutica* 2012, **163**(5):e339-348.

19. Markina I: **La gripe A, en la Prensa espansola**. *Revista Latina de Communicacion Social* 2009, **64**(1–16).

20. Oh H, Hove T, Paek H, Lee B, Lee H, Song S: **Attention cycles and the H1N1 pandemic: a cross-national study of US and Korean newspaper coverage**. *Asian Journal of Communication* 2012, **22**(2):214–232.

21. Olowokure B, Odedere O, Elliot AJ, Awofisayo A, Smit E, Fleming A, Osman H: **Volume of print media coverage and diagnostic testing for influenza A(H1N1)pdm09 virus during the early phase of the 2009 pandemic**. *Journal of clinical virology : the official publication of the Pan American Society for Clinical Virology* 2012, **55**(1):75–78.

22. Ringel JS, Trentacost E, Lurie N: **How well did health departments communicate about risk at the start of the Swine flu epidemic in 2009?** *Health affairs (Project Hope)* 2009, **28**(4):w743-750.

23. Salathe M, Khandelwal S: **Assessing vaccination sentiments with online social media: implications for infectious disease dynamics and control**. *PLoS computational biology* 2011, **7**(10):e1002199.

24. Schwartz RD, Bayles BR: **US university response to H1N1: a study of access to online preparedness and response information**. *American Journal of Infection Control* 2012, **40**(2):170–174.

25. Tausczik Y, Faasse K, Pennebaker JW, Petrie KJ: **Public anxiety and information seeking following the H1N1 outbreak: blogs, newspaper articles, and Wikipedia visits**. *Health communication* 2012, **27**(2):179–185.

26. Tirkkonen P, Luoma-aho V: **Online Authority Communication during An Epidemic: A Finnish Example**. *Public Relations Review* 2011, **37**(Journal Article):172–174.
